# Zika virus dysregulates the expression of astrocytic genes involved in neurodevelopment

**DOI:** 10.1371/journal.pntd.0009362

**Published:** 2021-04-23

**Authors:** Muhammad Adnan Shereen, Nadia Bashir, Rui Su, Fang Liu, Kailang Wu, Zhen Luo, Jianguo Wu

**Affiliations:** 1 State Key Laboratory of Virology, College of Life Sciences, Wuhan University, Wuhan, China; 2 Guangdong Provincial Key Laboratory of Virology, Institute of Medical Microbiology, Jinan University, Guangzhou, China; Beijing Children’s Hospital, Capital Medical University, CHINA

## Abstract

Zika virus (ZIKV) is a kind of flavivirus emerged in French Polynesia and Brazil, and has led to a worldwide public health concern since 2016. ZIKV infection causes various neurological conditions, which are associated with fetus brain development or peripheral and central nervous systems (PNS/CNS) functional problems. To date, no vaccine or any specific antiviral therapy against ZIKV infection are available. It urgently needs efforts to explore the underlying molecular mechanisms of ZIKV-induced neural pathogenesis. ZIKV favorably infects neural and glial cells specifically astrocytes, consequently dysregulating gene expression and pathways with impairment of process neural cells. In this study, we applied a model for ZIKV replication in mouse primary astrocytes (MPAs) and profiled temporal alterations in the host transcriptomes upon ZIKV infection. Among the RNA-sequencing data of 27,812 genes, we examined 710 genes were significantly differentially expressed by ZIKV, which lead to dysregulation of numerous functions including neurons development and migration, glial cells differentiation, myelinations, astrocytes projection, neurogenesis, and brain development, along with multiple pathways including Hippo signaling pathway, tight junction, PI3K-Akt signaling pathway, and focal adhesion. Furthermore, we confirmed the dysregulation of the selected genes in MPAs and human astroglioma U251 cells. We found that PTBP1, LIF, GHR, and PTBP3 were upregulated while EDNRB and MBP were downregulated upon ZIKV infection. The current study highlights the ZIKV-mediated potential genes associated with neurodevelopment or related diseases.

## Author summary

Zika virus (ZIKV) infection causes serious neurological disorders of central and peripheral nervous system, and fetal brain development disorders including microcephaly. There are still uncovered explorations for the underlying molecular mechanism of ZIKV-infected pathogenesis. This study reveals a series of dysregulation of neuropathic genes mRNA and protein expression in mouse and human astrocytes upon ZIKV infection. As an ideal ZIKV infection model in mouse primary astrocytes (MPAs), RNA-seq was performed to profile transcriptome alteration by ZIKV infection. Bioinformatics analysis demonstrated the significant alterations of the 710 genes that were linked to glial cell differentiation and projection, neurogenesis and migration of neurons, myelination, as well as synaptic control. Among the top selected differentially expressed genes, such as PTBP1, LIF, GHR, PTBP3, EDNRB, and MBP, the mRNA and protein expressions were confirmed to identify the dysregulation of the transcriptome in MPAs upon ZIKV infection. Furthermore, ZIKV infection altered the mRNA and protein expression of these astrocytic genes involved in neurodevelopment in U251 cells following the analysis of the transcriptome. In conclusion, the alteration of astrocytic gene functions or associated-pathways suggest a novel clue of a mechanism involved in the ZIKV-induced neurodevelopment disorders.

## Introduction

Zika virus (ZIKV) belongs to the Flaviviridae family, *Flavivirus genus*, and is transmitted to humans through *Aedes* mosquitoes bite and sexual intercourse [1–3]. ZIKV infection causes Zika fever that results in serious health issues for being potentially associated with fetus neurodevelopment disorders such as microcephaly and other central and peripheral nervous systems (CNS/PNS) neurological conditions including myelitis, encephalitis, polyneuritis, and demyelinating polyneuropathy [4–6]. ZIKV was isolated in the Zika forest of Uganda in 1947 from a monkey and the anti-ZIKV neutralizing antibodies in human serum were first reported in 1952 [7]. The first link between neurological diseases and ZIKV infection was described in the French Polynesia during the ZIKV outbreak in 2013–2014 [8,9]. In the near past between 2015 and 2016, an increase in the microcephaly prevalence reported in some areas of Brazil confirmed ZIKV transmission [10]. However, different approaches have been made to reveal the underlying links of microcephaly and ZIKV infection during pregnancy [11,12]. Additionally, ZIKV infection is also responsible for other neurological and brain abnormalities [10,11,13–15].

Differentiation failure or loss of the neuronal progenitor cell growth impairs neural development to causes microcephaly [16]. An increase in the fetal malformation cases upon ZIKV infection (congenital ZIKV syndrome), such as microcephaly and hydrocephaly, placental insufficiency in utero, stillbirth, and spontaneous abortion, indicating the capacity of ZIKV to readily infect the developing human brain, were reported as ZIKV infection deleterious consequences [17]. Most of these diseases are linked to the problems that occur during fetus brain development or CNS/PNS function [6]. To date, no antiviral therapy or vaccines are available for zika virus infection [18]. As a result, the relationship between neural disorders and ZIKV infection is of high concern.

Besides neural cells, glial cells such as microglia and astrocytes are the significant target cells for ZIKV infection in brain [19–22]. Glial cells are rich in astrocytes and found in the capillary’s vicinity as an important component of the blood-brain barrier (BBB). Thus, ZIKV could target astrocytes instantly after invading the CNS. Upregulation of pro-inflammatory molecules production upon Toll-like receptor (TLR) pathway activation in glial cells is a vital factor for neuro-inflammation which is associated with many other neurological disorders [23]. As such, ZIKV infection in glial cells may cause BBB leakage by producing pro-inflammatory molecules which leads to neuro-inflammation [24]. In addition, as astrocytes are exceedingly involved in synaptic and axonal guidance, other microglia’s coordination in postnatal synaptogenesis and neurons survival to death with genetic and functional alterations may result in various neurological disorders [25,26]. Astrocytes from different brain regions convert or help in-migration of new neuron through the rostral migratory stream organization during early developmental stages [25,27,28]. Based on this, the ZIKV infection in astrocytes is considered to be an important site in pathological neural disorders and brain abnormalities.

Emerging studies have been reported on virus-induced variations in the transcriptomic profiles of virus-treated cell lines and organisms [29,30]. Phenotypically, transcriptome profiling of ZIKV infection in mouse cortical tissues, human brain organoids, and neural progenitors have disclosed the dysregulation of many genes and pathways that are linked with cell death, DNA repair or replication, cell cycle, viral response, metabolism, and transcription [31–33]. The dysregulation of transcriptomes by ZIKV varies from Dengue virus (DENV) infection induced dysregulation [34]. ZIKV infection in embryonic mouse cortex and human neural progenitors dysregulate a number of known microcephaly-associated genes such as MCPH1 (Microcephalin), CENPF (Centromere Protein F), TBR2 (T-box brain protein 2), RBBP8 (RB binding protein 8), AXL (AXL receptor tyrosine kinase), and ASPM (Assembly factor for spindle microtubules) [31,35]. However, it remains unclear how such dysregulation may contribute to ZIKV-related pathophysiology in astrocytes.

This study evaluated the alteration of neuropathic genes mRNA and protein expression in mouse primary astrocytes (MPAs) and human astroglioma U251 cells upon ZIKV infection. We conducted an ideal ZIKV infection model in MPAs isolated from neonatal mice, followed by RNA-seq performance to profile ZIKV-induced transcriptome alterations. Briefly, we identified 710 significantly dysregulated genes from the RNA-seq of total of 27,812 genes upon ZIKV infection in MPAs, which were associated with astrocytic cell synaptic control, migration, cell growth and other brain development conditions under bioinformatic functional analyses. Subsequently, the most differentially dysregulated genes associated with neural function in MPAs were selected in ZIKV-infected MPAs. In detail, we selected top genes from the dysregulated pool and identified that LIF (Leukemia inhibitory factor), PTBP3 (Polypyrimidine tract binding protein 1), and GHR (Growth hormone receptor) were upregulated while EDNRB (Endothelin receptor type B) and MBP (Myelin basic protein) were downregulated upon ZIKV infection in both MPAs and U251 astrocytes. Another member from PTB family called PTBP1 plays a critical role in astrocyte-neuron conversion during neurodevelopment [27], was also detected along with PTBP3. Our findings systematically revealed dysregulation in a cluster of neural genes by ZIKV infection in astrocytes, which provides novel clues for the mechanism involved in ZIKV-associated neurodevelopment disorders. Datasets presented in this study could be a critical source to understand the molecular pathogenesis of ZIKV-induced abnormalities in fetal brain, and to develop an effective therapeutic approach against ZIKV infection and its consequences.

## Materials and methods

### Animal study

Wild type (WT) C57BL/6J mice were purchased from the Hubei Provincial Center for Disease Control and Prevention (Wuhan, Hubei, China). The mice were housed under specific pathogen-free (SPF) conditions in individually ventilated cages.

### Ethics statement

The animal study was approved by the Institutional Review Board of the College of Life Sciences, Wuhan University and was conducted in accordance with the guidelines for the protection of animal subjects (permit number: WDSKY0201901).

### Isolation of MPAs

Postnatal day 3 (P3) mice were sacrificed by decapitation for isolation of cortical astrocytes or also called MPAs. Cerebral cortices were carefully isolated and after processing to dissociate to a single cell suspension, and seeded in T75 tissue culture flask prior coated with Poly-D-lysine at a dose of 50 *μ*g/ml, as previously reported [36]. After 7 to 8 days of incubation, confluent astrocytes monolayers were shaken on an orbital shaker for 40 minutes at 180 rpm to remove microglia cells, and then replaced culture medium following a second time shaking at 240 rpm for 6 hours to remove oligodendrocytes precursor cells (OPCs). Astrocyte monolayers were washed with phosphate buffer saline (PBS) and transferred into a new T75 cultural flask, and incubated for 12 to 14 days replacing fresh medium for every 2 to 3 days until usage [36]. The isolation of pure astrocytes and removal of other cells like microglia and OPCs was confirmed by a morphological overview of mix cortical cells at different time points after isolation and immunofluorescence microscopy against the astrocyte-specific marker GFAP [36].

### Cell lines and culture

Mouse primary astrocytes (MPAs) were isolated from WT C57BL/6J mice brain cortices as described above [36]. Human astroglioma (U251), African green monkey kidney epithelial cells (Vero) (#CCL-81), Human lung cancer cell line (A549), and *Aedes* albopictus mosquito cell line (C6/36) (#CRL-1660) were purchased from ATCC (Manassas, VA, USA). All these cells were cultured in DMEM (Gibco; Grand Island, NY, USA) supplemented with 10% FBS (Gibco) with100 U/ml Penicillin/Streptomycin (Gibco) at 37°C and 5% CO_2_, except that C6/36 cells were maintained in RPMI1640 (Gibco) at 30°C and 5% CO_2_ [37].

### Reagents

Rabbit anti- EDNRB (A2980), MBP (A11162), LIF (A1288), GHR (A14735) and β-actin antibodies were purchased from ABclonal Technology (Wuhan, China). Rabbit anti-PTBP1 (12582-1-AP) antibody was purchased from Proteintech Group (Rosemont, IL, USA). Rabbit anti-dsRNA [J2] (Ab01299) was purchased from Absolute Antibody (Wilton, UK). Rabbit anti-ZIKV-protein (GTX133312) was purchased from GeneTex (Irvine, CA, USA). Mouse anti-GFAP (E4L7M) was purchased from Cell Signaling Technology (Beverly, MA, USA).

### Zika virus propagation and isolation

ZIKV Asian strain isolate z16006 (GenBank accession number, KU955589.1) obtained from Institute of Pathogenic Microbiology, Center for Disease Control and Prevention of Guangdong (Guangzhou, China) as previously reported [37]. ZIKV was propagated in C6/36 cell line. Briefly, C6/36 cells were cultured in RPMI1640 containing 10% FBS and 1% Penicillin/Streptomycin solution. Cells were infected with ZIKV at an MOI (Multiplicity of Infection) of 0.1 (diluted in 1 ml RPMI1640). After incubation for 2 h at 32°C, 5% CO_2_, 4 ml fresh medium was added to the culture and continued the incubation for additional 4 days. Cell supernatants were harvested, centrifuged and filtrated using a 0.45 μm filter. The stock was aliquoted in 1.5 ml Eppendorf tubes for single time thawing [37]. The determination of virus titer was performed by serial dilutions for the infectivity in Vero and A549 cells detected by standard plaque assay and further amplified by TCID_50_ (50% Tissue Culture Infective Dose) [38–40].

### RNA-seq and bioinformatics analysis

MPAs were infected with ZIKV at MOI = 2 and harvested at 24 h p.i. The detailed methodology of the transcriptomic study is present in S1 Text. Briefly, the total RNA samples were extracted in TRIzol reagent and cDNAs were prepared. The library preparations (S1 Table) were sequenced on an Illumina Hiseq 2500 PE150 platform (Illumina; San Diego, CA, USA) in Novogene Bioinformatics Institute (Beijing, China). The raw reads of RNA-seq sequences are treated by the fastp (https://github.com/OpenGene/fastp). Clean reads are aligned to the hg38 genome with HISAT2 [41], duplicates are removed using Samtools [42]. Genes identified as rRNAs, and pseudogenes are removed. Transcript assembly is determined using StringTie and Ballgown is used to identify differentially expressed isoforms gene [43]. Then, the differential expression analysis is performed using DESeq2 (https://bioconductor.org/packages/release/bioc/html/DESeq2.html), using a fold change (FC) cutoff of 1.5 and padj-value cutoff of 0.05. Gene set enrichment analysis was done using QuSAGE [44]. The gene symbols were converted to ensemble IDs using BioDBnet (https://biodbnet-abcc.ncifcrf.gov/db/dbOrthoRes.php). For expression-based analysis the data were subjected to an online ImageGP tool for converting numerical data into heatmaps (http://www.ehbio.com/ImageGP/index.php/Home/Index/PHeatmap.html), and volcano plot (http://www.ehbio.com/ImageGP/index.php/Home/Index/Volcanoplot.html). The significantly differentially expressed genes selected were subjected to functional annotation analysis (gene ontology) using online ToppGene Suit (https://toppgene.cchmc.org/enrichment.jsp) and DAVID Bioinformatics Resources 6.8 (https://david.ncifcrf.gov/summary.jsp) to find the most related KEGG pathways. The scripts (transcriptomic data) are available online in the NCBI Sequence Read Archive (SRA) database with the SRA (BioProject) accession number: PRJNA692303.

### RNA quality and quantity, relative quantitative real-time PCR

Total cellular RNA was extracted from mock and ZIKV-infected MPAs and U251 cells using TRIzol reagent (Invitrogen; Carlsbad, CA, USA), following the manufacturer’s instructions. A detailed explanation of methodology is provided in S2 Text. Briefly, the quality of extracted RNA was assessed by spectrophotometry (NanoDrop; Thermo Scientific) (A260/A280) [45,46] and cDNAs were prepared using HiScript II Q Select RT SuperMix, according to the manufacturer’s instructions. (http://www.vazymebiotech.com/products_detail/productId=80.html). Relative quantitative Real-time polymerase chain reaction (RT-qPCR) analysis was performed on Light Cycler 480 (Roche, Basel, Switzerland) using SYBR Green Real-time PCR master mix (Bio-Rad; Hercules, CA, USA) as previously described [45,47]. Briefly, in a reaction mixture of 10 μl SYBR, 1 μl cDNA diluted template, 1 μl specific gene primers (0.5 μl forward and reverse primer, 10 μM each), and RNase-free water to complete 20 μl as a final volume. The amplification protocol used as follows: denaturation at 95°C for 30 s, 40 cycles of 95°C for 5 s, 55°C for 30 s, and 72°C for 30 s), and a final extension step. The melting curve was obtained by heating the amplicon from 65°C to 95°C at 0.5°C s^−1^. The relative expression level was based on 3 biological replicates mean at each time point using the 2^−ΔΔCT^ approach [48]. *GAPDH* was used as an internal reference control in this study. Real-time PCR primers were designed by Primer Premier 5.0 (Premier Biosoft; Palo Alto, California, USA), and the sequences are provided in Table 1.

**Table 1:** Information of primers sequences for qPCR assay in this study.

Primer name	Sequences (5’ to 3’)	
	Human	Mouse
qRT GAPDH F	ACCCAGAAGACTGTGGATGG	GGGATGATGGAGGACGTGAT
qRT GAPDH R	TCAGCTCAGGGATGACCTTG	CCAATACGGCCAAATCCGTT
qRT ZIKV NS5 F	GGTCAGCGTCCTCTCTAATAAACG	GGTCAGCGTCCTCTCTAATAAACG
qRT ZIKV NS5 R	GCACCCTAGTGTCCACTTTTTCC	GCACCCTAGTGTCCACTTTTTCC
qRT LIF F	ATCTGTCCATCCCAACAGCA	TGGAGCTGTATCGGATGGTC
qRT LIF R	ATCCTGGACAAGGGTGAGTG	GCATTGAGCTTGACCTGGAG
qRT EDNRB F	AGATGTGTAAGCTGGTGCCT	TTAGCCCTGTGTTCGTCACT
qRT EDNRB R	AACCACAGAGACCACCCAAA	GTTAAAGCTCTCGGGCTTGG
qRT GHR F	ACTCAGCAGCCCAGTGTTAT	AGCGAAGTCCTCCGTGTAAT
qRT GHR R	ATATGGGCAGCTTGGTGAGT	GGATCCTCTGAAGCTGGTGA
qRT MBP F	GAAGGCCAGAGACCAGGATT	ACTTGCCATCCATCCTGTGA
qRT MBP R	TGAATCCCTTGTGAGCCGAT	CCGGATCCCTGAGTTCTCTC
qRT PTBP1 F	AGGTCACCAACCTCCTGATG	TCTAAGTTTGGCACCGTCCT
qRT PTBP1 R	GGGTCACCGAGGTGTAGTAG	TGAGACTGGTGAGCTTGGAG
qRT PTBP3 F	CAGATGGCGGATGCAAATCA	CCATCGCATTTCCTCAAGCA
qRT PTBP3 R	TGGTCTTCTTGTCCCTCTCG	AATGGCCATCCTTCCACTGA

qRT: quantitative Real-time PCR; F: forward; R: reverse.

### Western blot

Cell lysates were extracted in cell lysis buffer as previously described [49] (https://www.novusbio.com/support/support-by-application/western-blot/protocol.html). The protein concentrations were determined by Bradford assay (Bio-Rad, Hercules, CA, USA), subjected to 12% SDS-PAGE gel and transferred onto a nitrocellulose (NC) membrane. The membrane was blocked with 5% nonfat dried skim milk and incubated with specific antibodies. Protein bands were detected by using enhanced chemiluminescence Luminescent Image Analyzer (Fujifilm LAS-4000, Fujifilm, Tokyo, Japan). β-actin was used as an internal control refers to the target genes [37] (http://docs.abcam.com/pdf/protocols/general-western-blot-protocol.pdf).

### Bright-field microscopy

Morphological representation of U251 cells treated with ZIKV and Mock were observed for cytopathic effect (CPE) at 12, 24, 48, and 60 h p.i. using bright field Nikon TE-2000 inverted microscope (Tokyo, Japan). All images’ brightness and contrast were adjusted in PowerPoint 2016 [6].

### Cell viability

Cell viability was determined using the Cell Counting Kit 8 (CCK8) according to the manufacturer’s instructions (Dojindo, Japan). ZIKV-treated cells for indicated times were provided with 500 μl of CCK8 solution in each well in 12-well plate and incubated for 30 minutes at 37°C. Cell injury absorbance was measured at 450 nm [6,47].

### Confocal microscopy

The confocal microscopy was performed according to the method previously reported [6]. Briefly, MPAs and U251 cells were seeded on 20-mm, and mock-infected or with ZIKV- infected for different time intervals. The cells were washed with PBS, fixed with 4% formaldehyde for 15 min and then permeabilized with 0.2% Triton X-100 for 5 min at room temperature (RT). After three times wash with PBS, the cells were blocked with 5% bovine serum albumin (BSA) for 1 h, subsequently incubated with specific primary antibodies overnight at 4°C. The cells were washed and incubated with FITC-conjugated or Cy3-conjugated goat anti-rabbit IgG, FITC-conjugated goat anti-mouse IgG (Proteintech Group) secondary antibodies for 1 h at RT. Finally, the cells were washed and mounted for Immunofluorescence (IF) assay by confocal laser scanning microscopy (Fluoview FV1000; Olympus, Tokyo, Japan) after nuclei staining with 4,6-diamidino-2-phenylindole solution (DAPI) [6,37].

### Statistical analysis

All experiments were repeated at least three times. The results are presented as means ± SD unless stated otherwise. Statistical significance was determined by paired Student’s *t*-test using the Prism 8 software (GraphPad Software Inc., San Diego, CA, USA). A *P* ≤ 0.05 was considered statistically significant.

## Results

### The replication of ZIKV in MPAs

**Fig 1:**
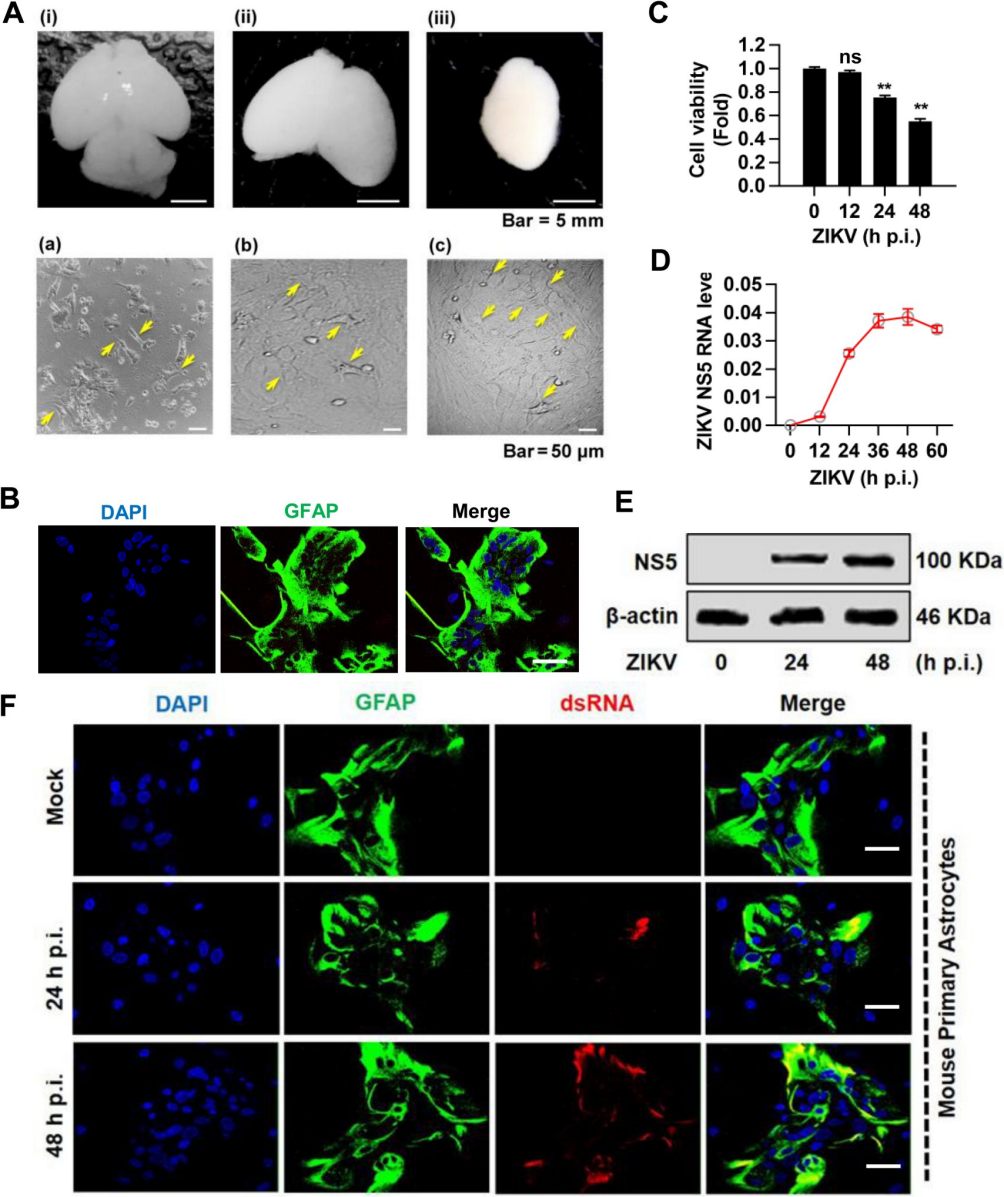
The isolation, culture, and identification of ZIKV infection in mouse primary astrocytes (MPAs). **(A)** Postnatal (P3) mouse dissection for cortical astrocytes isolation. (i) Whole brain; (ii) Brain after cerebellum and olfactory bulbs removal; (iii) Cortices isolation by peeling off the meninges, a plate-like structure on the brain cortex, scale bar 5 = mm. Culture of mouse primary astrocytes from mouse brain cortex. (a) The yellow arrows indicate single cell astrocytes attached to the bottom of the culture flask and floating are the dead neurons in supernatant. (b) Astrocytes layer is forming and confluent after vigorous shaking to remove OPCs and Microglia cells. (c) Primary astrocytes layer shows a high density of the cells after 2 weeks of the first split, scale bar = 50 μm. **(B)** Identification of mouse primary astrocytes with rabbit anti-GFAP (green) marker and DAPI (blue) for nuclei by confocal microscopy, scale bar = 20 μm. **(C-F)** MPAs were mock-treated or infected with ZIKV (MOI = 2) for indicated times. Cell viability was measured by CCK8 assay **(C)**, ZIKV viral NS5 mRNA **(D)** and NS5 protein expression (**E**) were determined by qPCR with *GAPDH* as an internal control and Western blot with β-actin as internal control, respectively, and viral dsRNA (a replication intermediate) was immunoprobed with dsRNA-antibody (red), along with GFAP (green) for astrocytes and DAPI (blue) for nuclei by Immunofluorescent (IF), scale bar = 20 μm **(F)**. The underlying numerical data for “Fig 1C and 1D” is provided in the supporting file “S1 Data”.

Considering astrocytes as one of the most significant target cells for ZIKV infection in the brain, we assessed the infection and replication of ZIKV in MPAs. Initially, we isolated and cultured MPAs from mouse brain cortex by standard protocol (Fig 1A). To confirm the purified isolation of MPAs, the cells were stained with astrocyte marker GFAP (glial fibrillary acidic protein). Immunofluorescence (IF) staining results revealed that nearly all isolated cells were GFAP positive (Fig 1B). We further evaluated ZIKV infection and replication in MPAs. The increasing cytopathic effect (CPE) was measured by cell viability assays (Fig 1C), ZIKV replication was determined by an obvious increase of viral NS5 RNA level detected by qPCR (Fig 1D), while expression of viral NS5 protein detected by Western blot (Fig 1E), suggesting a robust viral replication in MPAs upon ZIKV infection. Furthermore, IF analysis showed the ZIKV replication by the continuous increase of viral dsRNA in MPAs at 24 and 48 h p.i. (Fig 1F). Overall, these results illustrated that MPAs are susceptible to ZIKV infection and elicited well-observed replication, which allows MPAs as a kind of considerable model for ZIKV infection.

### Profile of mRNA expression in ZIKV-infected MPAs

**Fig 2:**
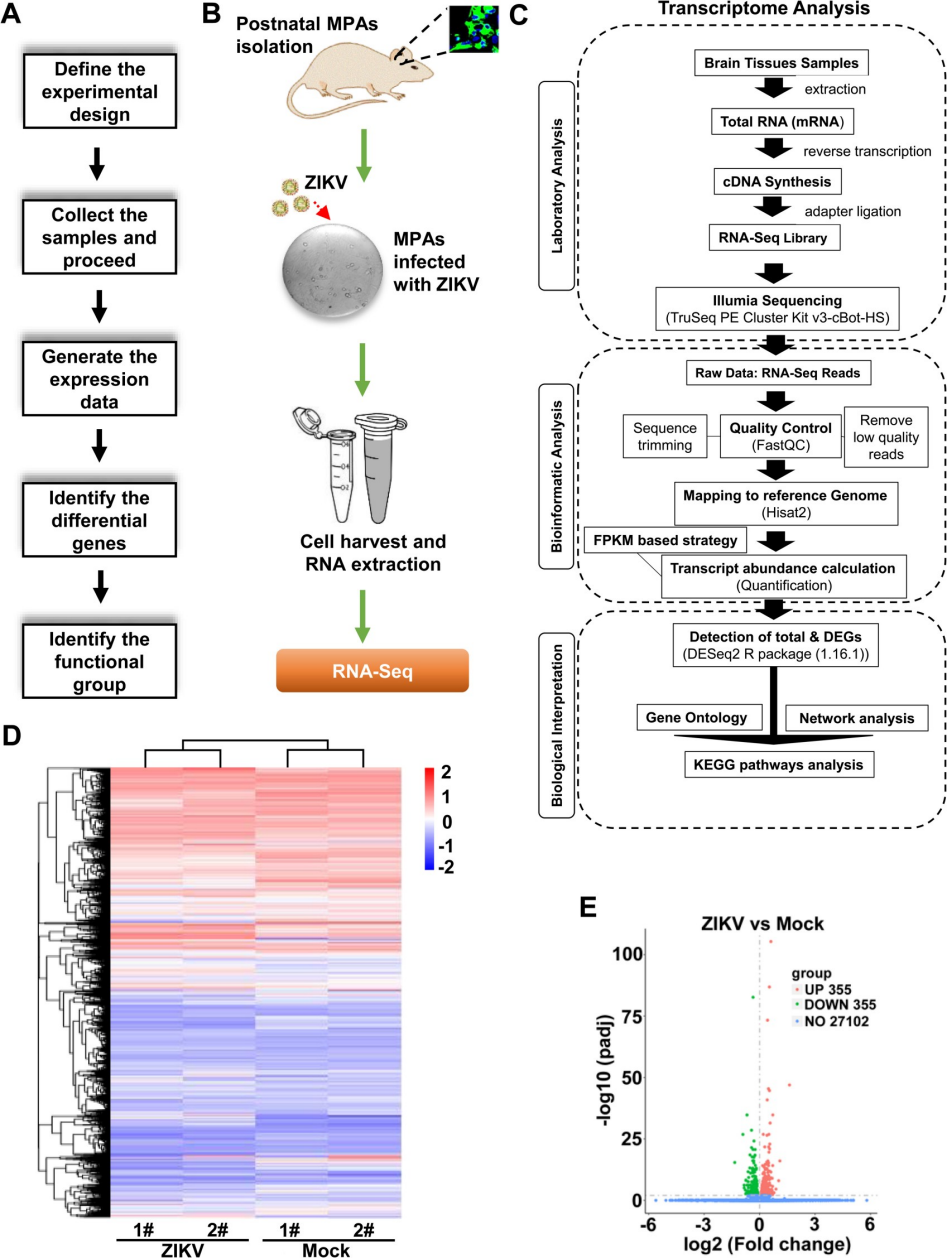
Expression profile for altered mRNAs upon ZIKV infection in MPAs. **(A**) A basic workflow diagram for the differential gene expression profile starting from experimental design to using bioinformatic tools. (**B**) Sketch diagram of main experiments from isolation of MPAs to RNA-Seq. (**C**) Flow sheath of transcriptome analysis of MPAs upon ZIKV infection. (**D**) Hierarchical clustering Heatmap of a distinguishable mRNA expression profiling in mouse primary astrocyte cells of ZIKV-infected group relative to Mock group (each group, n = 2). (**E**) Volcano analysis for a profile of mRNAs expressions of ZIKV-infected relative to Mock group. Red dots illustrate up-regulated genes, green dots represent down-regulated genes, while blue dots show insignificant genes. The underlying numerical data for “Fig 2D and 2E” is provided in the supporting file “S1 Data”.

To explore the transcriptional differences by ZIKV infection in MPAs, RNA-Seq was performed and the transcriptional profile for altered genes was designed (Fig 2A). Briefly, we cultured MPAs isolated from the mouse brain cortices and then infected them with ZIKV, following cell harvest and RNA-Seq (Fig 2B). The transcriptome analysis was proceeded according to laboratory analysis, bioinformatic analysis, and biological interpretation (Fig 2C). In total 27,812 genes represented by a heatmap (Fig 2D), we identified 710 significantly dysregulated genes upon ZIKV infection in MPAs based on fold change with cut off value of 1.5 (S2 Table). Among these 710 genes, 355 genes were significantly up-regulated and 355 genes were down-regulated upon ZIKV infection in MPAs (Fig 2E). Thus, the data demonstrated that ZIKV infection altered transcriptome in MPAs.

### Functional categorization of the differentially expressed genes by ZIKV in MPAs

**Fig 3:**
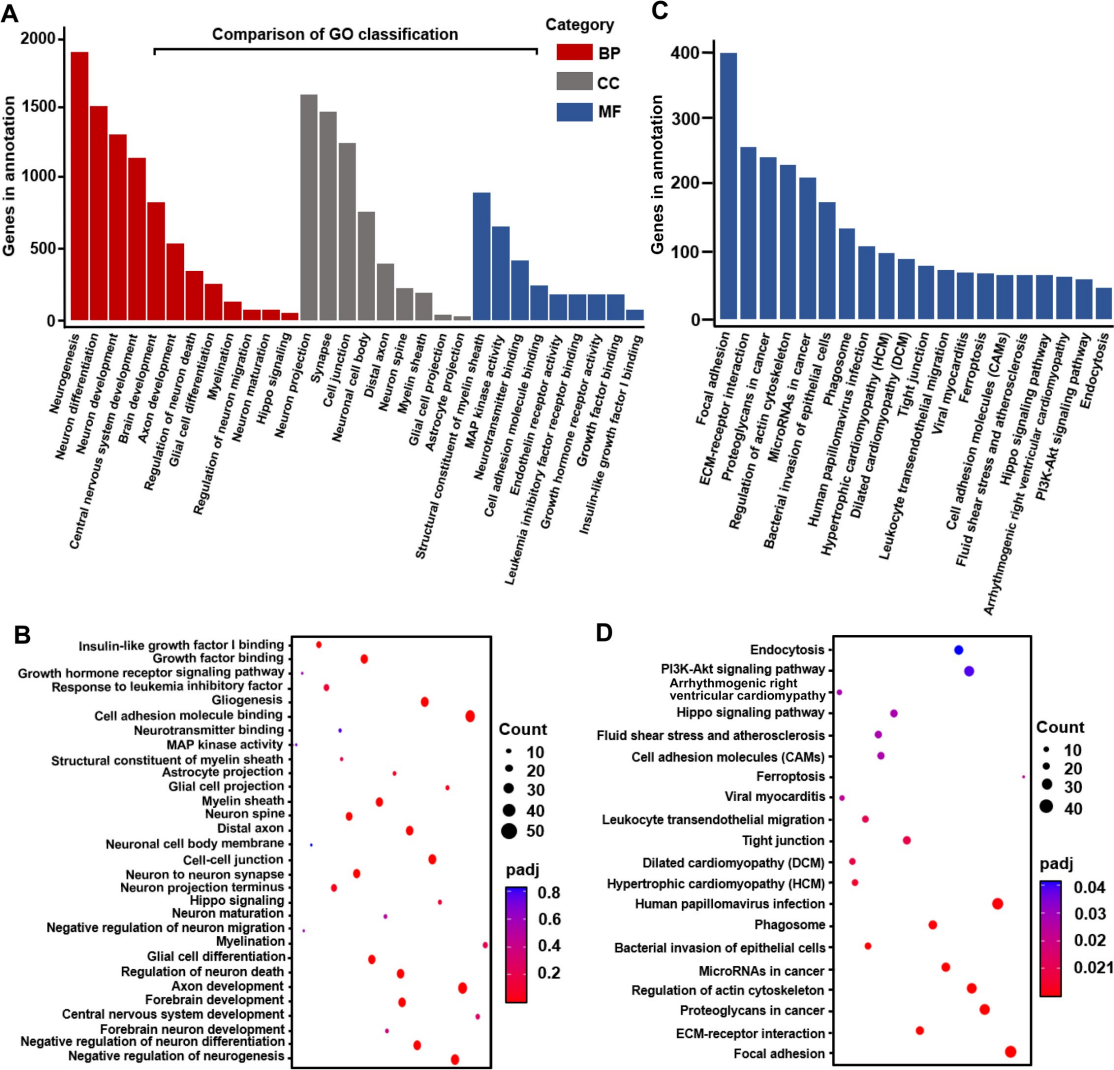
Functional categorization of the differentially expressed genes (DEGs) in ZIKV-infected MPAs. **(A)** The DEGs were assigned to three gene ontology classes: biological process (BP, red color), cellular component (CC, dark grey color), and molecular function (MF, blue color), with top 30 functional terms. **(B)** The dot plot displays the enrichment score values of the top 30 most significant enrichment terms. **(C)** Pathway enrichment (top 20) involved in significant genes reported in ZIKV-infected relative to Mock mouse primary astrocyte cells from RNA-seq analysis. KEGG was used as the pathway database. Enrichment scores are based on (A). **(D)** Top 20 enriched KEGG pathways among the annotated DEGs in 2 groups comparisons. The Y-axis represents KEGG pathways. Low padj values are shown in red, and high padj values are depicted in blue. Pathways with padj values less than 0.05 are significantly enriched. The size of the spot reflects the number of DEGs, and the color of the spot corresponds to different padj-value ranges. The underlying numerical data for “Fig 3A, 3B, 3C, and 3D” is provided in the supporting file “S1 Data”.

Transcriptome analyses provide a better understanding of how the genome is transformed into functional proteins. ZIKV-treated human brain organoids, neural progenitors, and mouse cortical tissues including primary astrocytes exposed numerous altered gene ontologies or functions and pathways associated with cell death, metabolism, transcription, DNA replication and repair, cell cycle, and viral responses [50]. Since the transcriptome alteration was a conspicuous molecular phenotype of ZIKV infection in MPAs, we further showed the top 30 dysregulated basic functions of the related genes, and assigned the altered genes ontologies to three classes: biological process (BP, red color), cellular component (CC, dark gray color), and molecular function (MF, blue color) as reported previously [51], with 30 functional terms and genes in the annotation (Fig 3A). The dot plot also showed the enrichment score values of the top 30 most significant enrichment terms (Fig 3B), suggesting that ZIKV induces a change in the expression of genes associated with neurogenesis, neuron differentiation, development, migration, and maturation, astrocytes projection, and brain development in infected cells.

Furthermore, we showed the pathway enrichment results (top 20) involved in significant genes between mock and ZIKV-infected MPAs from RNA-seq analysis. KEGG was used as the pathway database and enrichment scores was depicted (Fig 3C). The comparisons of top 20 enriched KEGG pathways among the annotated differentially expressed genes in 2 groups were shown (Fig 3D), which reveals that ZIKV infection was related to multiple pathways including Hippo signaling pathway, focal adhesion, PI3K-Akt signaling pathway, and tight junction. Thus, the functional categorization is associated with brain development, and neurogenesis from differentially expressed genes by ZIKV in MPAs.

### ZIKV infection dysregulate neural genes expression in MPAs

Bioinformatic analysis of RNA-seq data illustrated that ZIKV can potentially dysregulate the astrocyte genes. We summarized the top 10 genes that were significantly differentially expressed (up- and down- regulated) among the 710 genes, which were significantly dysregulated in ZIKV-infected MPAs at 24 h p.i. compared to mock-treated cells. The comparisons of gene expression between two groups are presented by fold change (Table 2). It is known that these selected genes were directly or indirectly involved in neurological conditions or brain development [52,53], which is consistent with the fact that ZIKV infection dysregulates a number of known genes in the embryonic mouse cortex and human neural progenitors that are associated with neurodevelopment or neural disorders [31].

**Table 2:** The enrichment of top 10 genes in ZIKV infected MPAs.

List of most variable differentially expressed genes from RNA-seq data. (FC ≥ 1.5, P < 0.05)							
Expression	NCBI ID	Gene ID	Description	ZIKV	Mock	log2(V/M)	*P*-values
Up-regulated	14528	ENSMUSG00000037580	GTP cyclohydrolase 1	411.0951	255.2346	0.686851214	2.80E-05
	16948	ENSMUSG00000024529	lysyl oxidase	1955.848	1278.814	0.611777167	1.96E-14
	14600	ENSMUSG00000055737	growth hormone receptor	971.2079	693.0997	0.485122031	1.40E-05
	16878	ENSMUSG00000034394	leukemia inhibitory factor	821.1017	591.2651	0.475197313	6.50E-05
	19878	ENSMUSG00000020580	Rho-associated coiled-coil containing protein kinase 2	5737.706	4195.744	0.45306051	7.90E-19
	17294	ENSMUSG00000051855	mesoderm specific transcript	1454.704	1083.248	0.427033195	1.43E-06
	20393	ENSMUSG00000019970	serum/glucocorticoid regulated	1304.022	974.9833	0.418896285	9.53E-06
	230257	ENSMUSG00000028382	polypyrimidine tract binding protein 3	1069.396	808.1253	0.40267405	9.09E-05
	66234	ENSMUSG00000031604	methylsterol monooxygenase 1	2787.93	2108.884	0.400968214	2.97E-09
	19205	ENSMUSG00000006498	polypyrimidine tract binding protein 1	4517.417	4254.308	0.085671	0.084453
Down-regulated	17196	ENSMUSG00000041607	myelin basic protein	621.6153	945.9696	-0.607703751	1.71E-07
	50913	ENSMUSG00000039830	oligodendrocyte transcription	1866.69	2697.02	-0.53178784	2.98E-16
	17172	ENSMUSG00000020052	achaete-scute family bHLH transcription factor 1	321.8875	465.8106	-0.53166219	0.000573
	17762	ENSMUSG00000018411	microtubule-associated protein tau	685.4954	958.5018	-0.483424535	3.92E-06
	18823	ENSMUSG00000031425	proteolipid protein (myelin) 1	549.6362	767.9758	-0.482498168	3.89E-05
	13618	ENSMUSG00000022122	endothelin receptor type	5027.983	6649.826	-0.40246941	6.08E-21
	18128	ENSMUSG00000026923	notch 1	3787.42	4951.807	-0.387878131	1.88E-15
	23805	ENSMUSG00000020135	adenomatosis polyposis coli 2	2954.399	3839.891	-0.378530646	1.38E-12
	20847	ENSMUSG00000040033	signal transducer and activator of transcription 2	2639.62	3375.84	-0.354998851	5.01E-10
	12266	ENSMUSG00000024164	complement component 3	204812	261725.1	-0.353735272	6.43E-87

ENSMUXXXX, ensemble gene IDs; V, MPAs with ZIKV infection; M, MPAs with mock infection; FC, fold change

**Fig 4:**
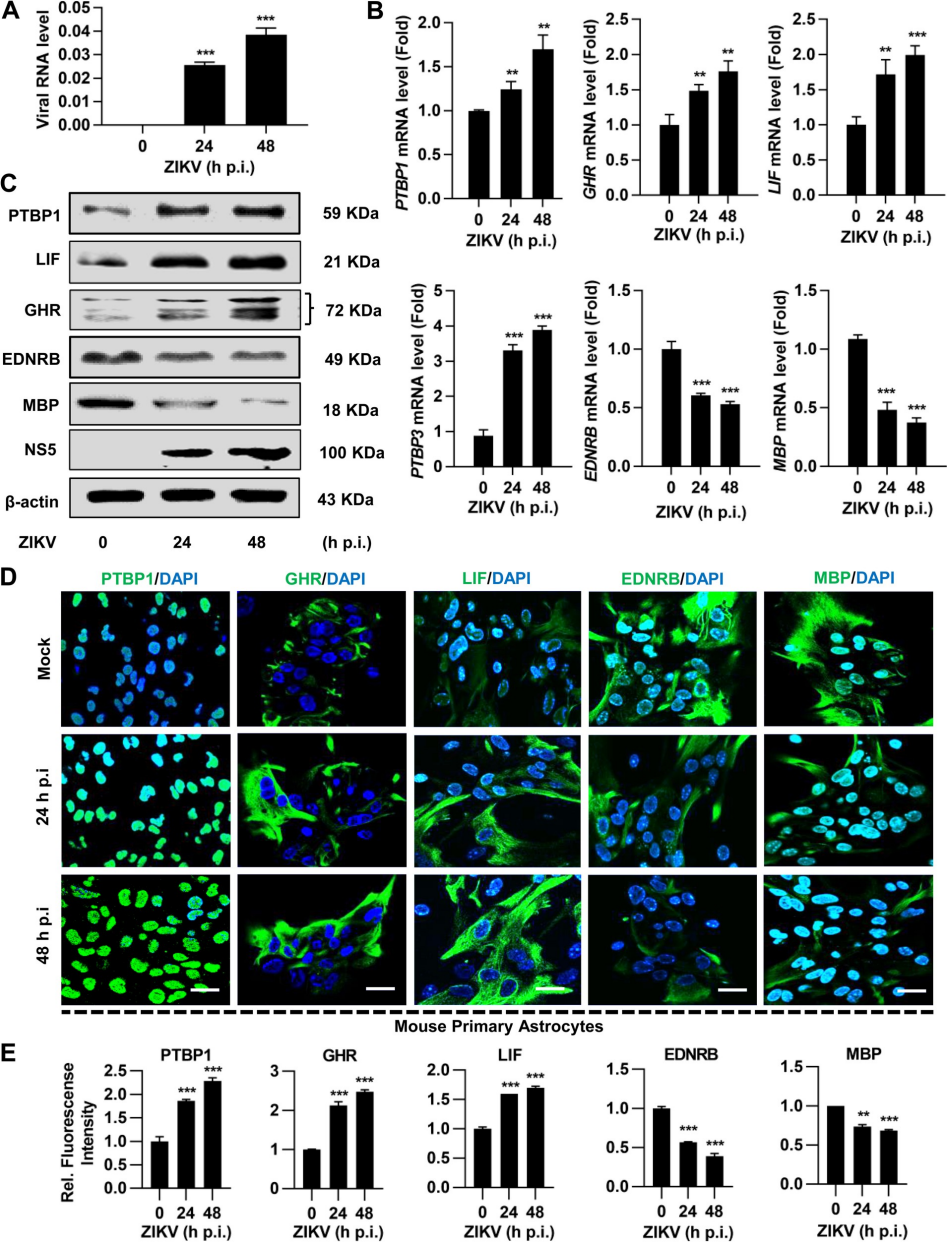
ZIKV infection dysregulates certain astrocytic genes in MPAs. MPAs were infected with ZIKV at MOI of 2 for 0, 24, and 48 h, respectively. **(A)** ZIKV NS5 RNA was determined by qPCR analysis in MPAs. **(B)** The mRNA expressions of *PTBP1*, *GHR*, *LIF*, *PTBP3*, *EDNRB*, and *MBP* were determined by qPCR. The data represent the relative expression of target genes normalized to *GAPDH* as a reference internal control. (**C**) The protein expressions of PTBP1, LIF, GHR, EDNRB, and MBP were determined by Western blot analyses. The β-actin was selected as an internal control. **(D** and **E)** Confocal microscopy images from MPAs stained with PTBP1, GHR, LIF, EDNRB, and MBP (green) for target genes and DAPI (blue) for nuclei, scale bar = 20 μm (**D**). Relative protein expression to control is quantified using Image J software **(E)**. Data are shown as fold change from mock control. All the experiments were performed in triplicate, non-significant (ns); *P* < 0.05 (*); *P* < 0.01 (**); *P* < 0.001 (***). Student’s *t*-test. The underlying numerical data for “Fig 4A, 4B, and 4E” is provided in the supporting file “S1 Data”.

Since ZIKV infection is responsible for causing differential gene expression in MPAs, we further selected six cellular genes that were significantly dysregulated by ZIKV infection to validate the RNA-seq results. ZIKV RNA (Fig 4A) and protein (Fig 4C) expression levels for NS5 were detected to confirm the significant infectivity and replication at 24 and 48 h p.i. We determined the differential gene expression in MPAs by qPCR. Our results showed that mRNA levels of *PTBP1*, *LIF*, *PTBP3*, and *GHR* were significantly upregulated, while *EDNRB* and *MBP* were downregulated upon ZIKV infection at indicated time (Fig 4B). The similar results were also observed for the protein expression of *PTBP1*, *LIF*, *GHR*, *EDNRB*, and *MBP* genes (Fig 4C). Furthermore, IF assay (Fig 4D) along with the intensity of illustrated gene expression (Fig 4E) confirmed the upregulation and downregulation of indicated gene expression in ZIKV-infected MPAs, respectively. These results suggested that ZIKV infection potentially dysregulated neural genes in MPAs, which may lead to neurodevelopmental disorders.

### Validation of DEGs in ZIKV-infected MPAs

**Fig 5:**
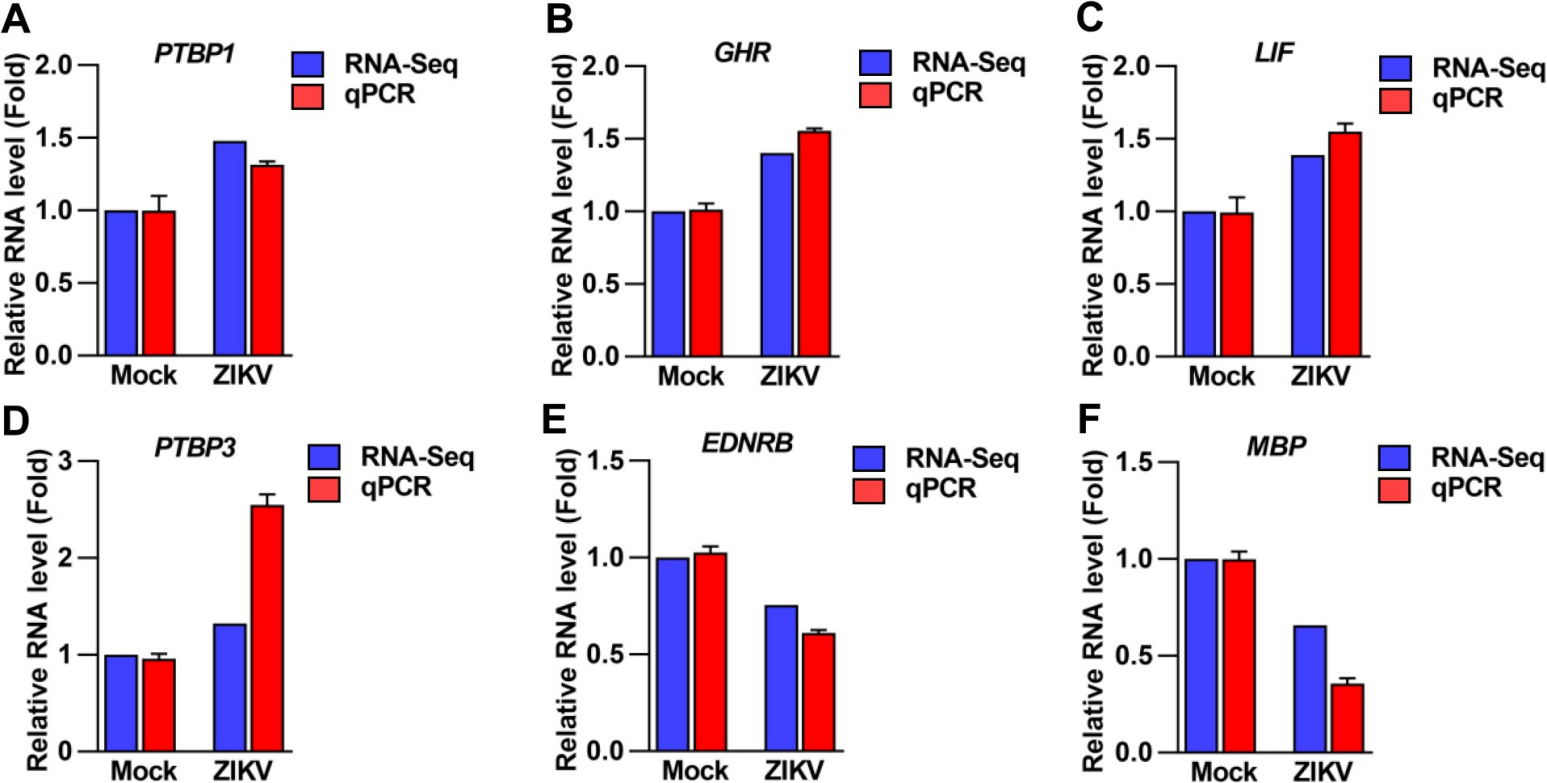
Validation of astrocytic altered genes upon ZIKV infection. MPAs were infected with ZIKV at MOI of 2 for 0 and 24 h, respectively. (**A**-**F**) The expression patterns of top 6 shortlisted genes: *PTBP1* (A), *GHR* (B), *LIF* (C), *PTBP3* (D), *EDNRB* (E), and *MBP* (F), were measured by RNA-seq and qRT-PCR (qPCR), respectively. *GAPDH* was used as an internal control in qRT-PCR. The individual bars represent the qPCR data as the means ± SD, n = 3. The underlying numerical data for “Fig 5A–5F” is provided in the supporting file “S1 Data”.

To access the validation of DEGs in ZIKV-infected MPAs, the comparison of the top 6 selected differentially expressed genes between qRT-PCR and RNA-Seq was performed. In the ZIKV-infected MPAs for 24 h, qRT-PCR analysis displayed the up-regulation of *PTBP1* (Fig 5A), *GHR* (Fig 5B), *LIF* (Fig 5C), and *PTBP3* (Fig 5D), while the down-regulation of *EDNRB* (Fig 5E) and *MBP* (Fig 5F), revealing that the selected genes expression profiles were accordant with obtained results from RNA-Seq (Fig 5A–5F). Therefore, the data illustrated that the methodology in the study concluded the accuracy and reproducibility.

### ZIKV infection dysregulate neural genes expression in human astroglioma U251 cells

**Fig 6:**
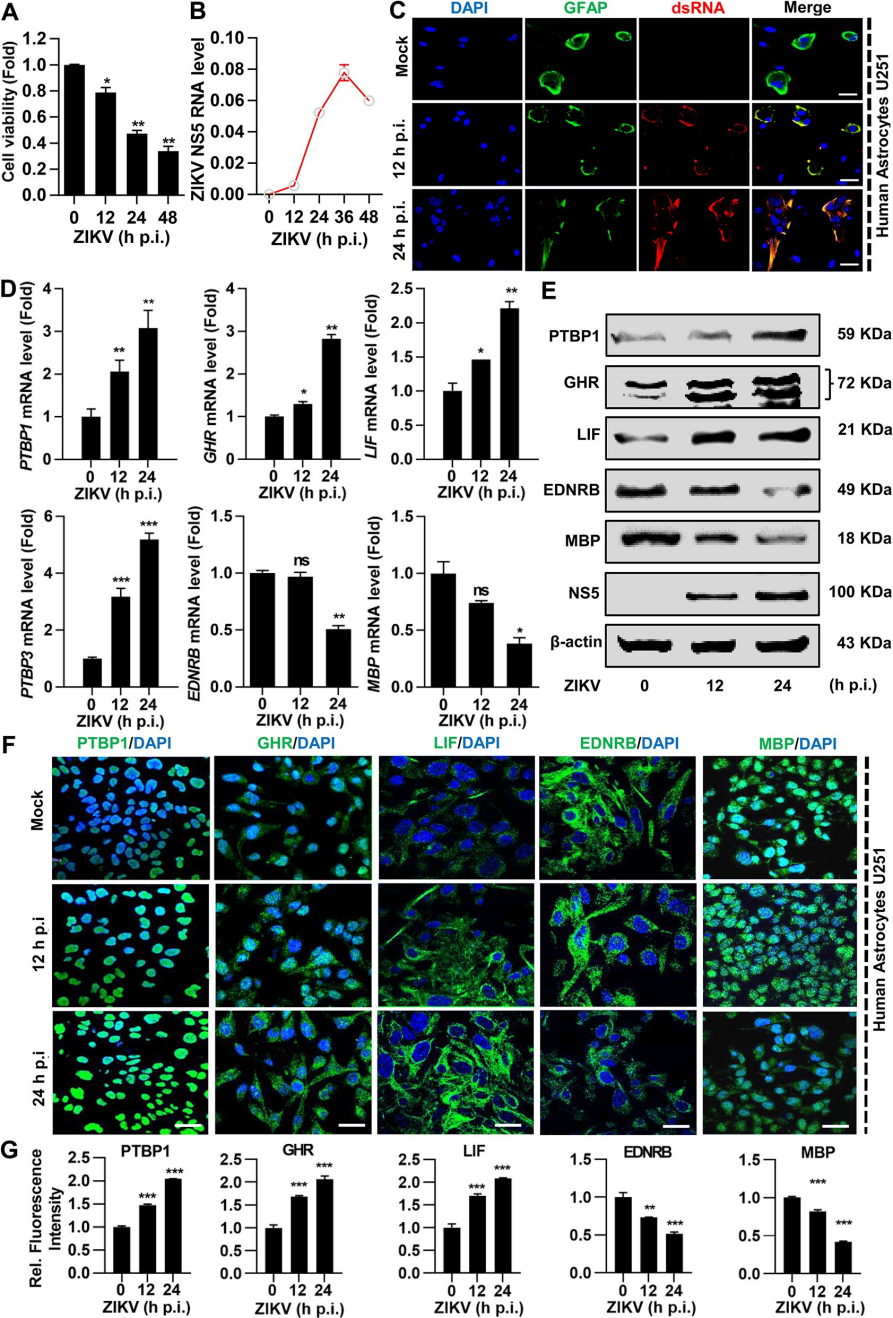
ZIKV infection differentially alters certain astrocytic genes in U251 cells. U251 cells were infected with ZIKV at MOI of 2 for indicated periods. **(A)** Cell viability was measured by the CCK-8 assay. Data represent as fold change compared to mock-treated cells. **(B)** ZIKV NS5 RNA was determined by qPCR with *GAPDH* as an internal control, and **(C)** viral dsRNA was immunoprobed with dsRNA-antibody (red), along with GFAP (green) for astrocytes and DAPI (blue) for nuclei by Immunofluorescent (IF), scale bar = 20 μm. **(D** and **E)** The mRNA expressions of PTBP1, GHR, LIF, PTBP3, EDNRB, and MBP were determined by qPCR (**D**) and protein expressions of PTBP1, GHR, LIF, EDNRB, and MBP was determined by Western blot analyses (**E**), respectively. In the qPCR analysis, the data represent the relative expression of target genes normalized to *GAPDH* as a reference internal control. In Western blot analysis, the β-actin was selected as an internal control. **(F** and **G)** Confocal microscopy images from MPAs stained with PTBP1, GHR, LIF, EDNRB, and MBP (green) for target genes and DAPI (blue) for nuclei, scale bar = 20 μm (**F**). Relative protein expression to control is quantified using Image J software **(G)**. Data are shown as fold change from mock control. All the experiments were performed in triplicate, non-significant (ns); *P* < 0.05 (*); *P* < 0.01 (**); *P* < 0.001 (***). Student’s *t*-test. The underlying numerical data for “Fig 6A, 6B, 6D, and 6G” is provided in supporting file “S1 Data”.

**Fig 7:**
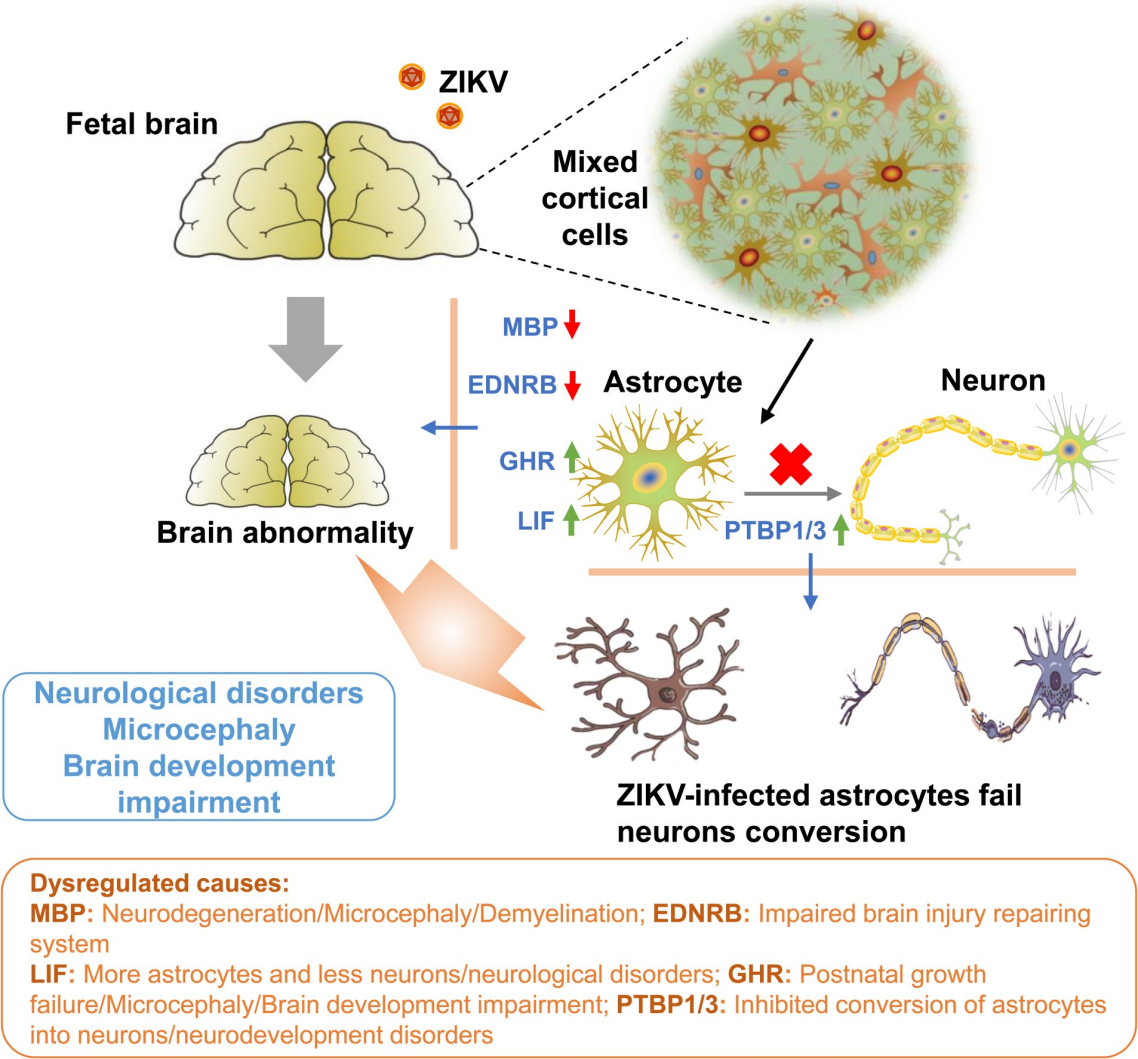
A mechanism underlying the brain-related neurological disorders upon ZIKV infection. The neurotropic ZIKV favorably infects and replicates in astrocytes of the fetal brain cerebral cortex. ZIKV can alter the astrocytic genes including PTBP1, LIF, PTBP3, GHR, EDNRB, and MBP, which are involved in various functions leading to neurological conditions such as microcephaly and brain development impairment. The numerical data used in all figures are included in S1 Data.

Given the fact that MPAs were susceptible to ZIKV infection and ZIKV dysregulated several critical neural genes expression, such alterations by ZIKV infection were also evaluated in human U251 cells. As compared to mock-treated U251 cells, ZIKV-infected U251 cells displayed a visible cytopathic effect (CPE) in a time-dependent fashion (S1 Fig), and the increasing CPE was measured by cell viability assays (Fig 6A). The qPCR results revealed that ZIKV RNA level continuously increased from 0 to 36 h p.i., whereas slightly declined between 36 and 48 h p.i. (Fig 6B), suggesting U251 cells were susceptible to ZIKV infection. In parallel, we also observed a robust expression of viral dsRNA upon ZIKV infection in U251 at 12 and 24 h p.i. (Fig 6C). Furthermore, we evaluated the differential gene expression of indicated genes in ZIKV-infected U251 cells. Our results illustrated that *PTBP1*, *LIF*, *PTBP3*, and *GHR* mRNA levels were upregulated while *EDNRB* and *MBP* mRNA levels were downregulated in ZIKV-infected U251 cells at 12 and 24 h p.i. (Fig 6D). Similarly, we also observed the dysregulated protein expression of the indicated genes by Western blot assay (Fig 6E) and IF assay (Fig 6F), along with the intensity of illustrated genes expression (Fig 6G). Notably, ZIKV infection dysregulated astrocytic gene expression leading to brain abnormality in human U251 cells as similar to MPAs (Fig 7), which suggests ZIKV-infected MPAs as a compatible infection model in the exploration of neural disorders.

## Discussion

An increase of defects in neurodevelopmental has been associated with ZIKV, a flavivirus mainly transmitted by mosquitoes and sexual transmission [54,55]. Current studies have revealed that ZIKV can infect human neurospheres, neural progenitor cells, and brain organoids [31,56–58]. However, the exact mechanisms of infection and specifically which cell populations in the nervous system respond to ZIKV and contribute to brain developmental disorders is still unclear. In current study, we used the RNA sequencing to analyze the astrocytic mRNAs that are dysregulated upon ZIKV infection. Utilizing different bioinformatic analysis coupled with gene expression profiling, our findings suggest that ZIKV can potentially alter the astrocytic gene expression, which is associated with brain development and neurological conditions [18]. Almost half or more of the brain cells are represented by glial cells (in specific parts up to 90%) and glial cell development is critical for normal brain size and function [59]. Astrocytes are the abundant and first line of the brain glia cells that are infected or respond to ZIKV infection [24]. Several studies have been reported on the association of ZIKV with brain development or associated diseases but yet there are no potential therapeutic options to overcome indicated neurological conditions [18]. For a developing brain, astrocytes are the drivers and initiators of ZIKV infection. Therefore, this virus not only infects brain cells and its precursors but also can penetrate the placental barrier and causes different neurological disorders linked to PNS and CNS [6].

Several studies reported the gene expression profiles in ZIKV-induced human and mouse neuronal cells [60–62], however, the association between the changes in astrocytic mRNAs expression and neurodegeneration or brain development is not described. In this study, we aimed to figure out the underlying link between the ZIKV and brain astrocyte cells. Initially, we constructed a ZIKV infection model in MPAs by isolating cortical astrocytes from the postnatal mouse brain, following ZIKV infection for indicated time points. ZIKV infection was confirmed in the MPAs and human astroglioma U251 cells, indicating these two kinds of astrocytes susceptible to ZIKV infection and considerable infection model in the exploration between ZIKV and astrocytes. At present, human neural progenitor cells [63], brain organoids [56,57,64] are the platform to study zika virus-associated microcephaly and embryonic brain development in vitro. Primary cell culture is increasingly being a major tool in virus and host interaction, which provides an excellent model for the study of normal physiology of the cells when exposed to virus infection [65]. Our study identified MPAs along with U251 cells behaving as a considerable tool to explore the relationship of ZIKV and host, which is consistent with evidence that ZIKV can target human glial cells [66,67].

We evaluated the ZIKV-induced MPAs for transcriptomics by RNA-seq and illustrated a huge number of astrocytic genes altered by ZIKV infection. Dysregulation of the neuronal genes, such as RBBP8, ASPM, AXL, TBR2, MCPH1, and CENPF upon ZIKV infection may cause different neurological or brain development related disorders [35]. By applying the bioinformatic tools, our RNA-seq data showed 27,812 genes alteration from MPAs upon ZIK infection, and around 355 genes were significantly dysregulated from each group of the differentiated the up- and down-regulated genes. Cellular function of the astrocytic genes, including modulation of stress response, remyelination, cell growth dysregulation, inhibition of differentiation, and modulation of stress response, have been identified in C6/36, Vero cells, human neural stem cells, and mouse glial cells [68,69]. The bioinformatic analysis revealed that ZIKV infection could interrupt neurogenesis, neuron differentiation, development, migration, and maturation, astrocytes projection, and brain development, as well as multiply pathways, including Hippo signaling pathway, focal adhesion, PI3K-Akt signaling pathway, and tight junction. To make the RNA-seq results more authentic, we further evaluated 6 genes associated with neurological disorders or brain development were significantly dysregulated upon ZIKV infection. Our results demonstrated that the expressions of PTBP1, PTBP3, LIF, and GHR were upregulated while EDNRB and MBP were downregulated in both ZIKV-infected MPAs and U251 cells, implying a potential dysfunction in ZIKV-induced neural disorders.

Among the selected differentially expressed genes, the RNA binding PTBP1 and PTBP3 from PTB family, which are responsible for the conversion of astrocytes into neurons [27], neurodevelopment including control of neuronal progenitor cells (NPCs) and embryonic stem cells (ESCs) splicing, and control of neural differentiation timing [70], are naturally decreased during neural progenitors’ differentiation [71] and give rise to multiple neuronal cell lineage in the brain [70,72]. On this account, our results suggest that the ZIKV-induced upregulation of the PTBP1 and PTBP3 levels in astrocytes may decrease or inhibit the conversion to neurons and eventually lead to various neurological conditions or make neurological infections more venerable.

LIF is a pleiotropic cytokine that promotes neurogenesis in fetus cerebrum [73,74] and autoimmune diseases treatment [75]. It is involved in neuroprotection, axonal regeneration, and preventing demyelination [76]. In addition, it induces neuronal progenitors [73] and acts as a stimulator for astrocytes differentiator [28]. Various studies reported that neurons and astrocytes are produced from the same precursor of CNS [28]. Based on these reports, our findings suggest that LIF works as a stimulator for astrocytes differentiation, so the upregulation of LIF may lead to the production of more astrocytes and fewer neurons, which results in different brain development defects.

We also reported the ZIKV-induced downregulation of EDNRB and MBP genes expression in astrocytes. EDNRB promotes reactive astrogliosis and helps in repairing brain injuries (remyelination) [77,78], while MBP releases from a sheath which plays a vital role in compacting myelin and protection of myelin sheath from degradation by myelinotoxic factors (proteinases) produced during multiple sclerosis [79], revealing that these two genes are essential housekeeping factors for normal myelin sheath control. Based on the previous studies, our results suggest that the downregulation of these genes might be the cause of neurological or brain disorders related to demyelination. Moreover, our results are consistent with the fact that the depletion or low EDNRB expression leads to defective B cell differentiation or intrinsic lymphoid defects [80], similar to that MBP reduction causes neurodegenerative disorders like microcephaly [81]. GHR promotes normal human postnatal growth and its deficiency or mutation may lead to utero and severe postnatal growth failure, intellectual impairment, microcephaly, and sensorineural deafness [82]. Additionally, upregulation of GHR levels have been reported to increase the risk of cancer in general and regulate key cellular functions, such as metastasis, apoptosis, proliferation, and differentiation [83]. The disruption of GHR also cause Laron syndrome (microcephalic syndrome) which not only affects the brain but also total somatic dwarfism [84,85]. In current study, the upregulation of GHR suggests that the alteration of this gene by ZIKV may lead to neurological impairments causing reported diseases, which further indicate that MBP and GHR are directly involved in the brain development and microcephaly.

Actually, there are some limitations in the study and some considerations should be taken into account when interpreting the findings. Firstly, the model we selected is the primary cells with some disadvantages, including limited potential for self-renewal and differentiation, which needs a further platform, such as human neural progenitor cells or brain organoids when studying ZIKV-associated microcephaly or embryonic brain development in vitro. Secondly, there should be more ZIKV strains included to access the astrocytic gene alteration to uncover the common rules of ZIKV-associated neurodevelopment. Thirdly, the dysfunctional genes associated with ZIKV-induced neurodevelopment are identified with observable expression regardless of poorly expressed genes, which may contribute to the uncertainty of findings. Even so, we verified a considerable model that ZIKV alters the transcriptome and discovered potential genes associated with neurodevelopment in MPAs, which requires an advanced model (e.g., human neural progenitor cells or brain organoids) and viral scope of ZIKV for biological functional exploration of neurodevelopment with genetic approaches (e.g., CRISPR-Cas9 or RNAi) in the future study.

In conclusion, the present study revealed ZIKV-infected MPAs as a compatible infection model for the exploration of ZIKV and astrocytes in neural disorders. In addition, we systematically exposed dysregulation in a cluster of neural genes by ZIKV infection in astrocytes, which provides novel clues for the mechanism involved in ZIKV-associated neurodevelopment or brain development impairment, and also lay the foundation for an approach to mechanistic research to develop potential vaccines or treatment against ZIKV infection to guard innumerable children and mothers exposed to the unfolding epidemics.

## Supporting information

S1 Fig The morphological study of ZIKV-infected U251 cells. U251 cells were infected with ZIKV at an MOI of 2 for indicated periods. The cytopathic effect (CPE) was displayed in U251 cells infected with ZIKV (bottom) compared to mock (top) with bright-field microscopy, scale bar = 50 μm.
(TIF)

S1 Text Transcriptome Analysis Methodology. (DOCX)

S2 Text qRT-PCR Methodology. (DOCX)

S1 Table Raw Data Information. (XLSX)

S2 Table List of All 710 DEGs. (XLS)

S1 Data Numerical Raw data for Figures. Excel spreadsheet in separate sheets contains the underlying numerical data for Figure panels 1C, 1D, 2D, 2E, 3A & 3B, 3C & 3D, 4A, 4B, 4E, 5A-5F, 6A, 6B, 6D, 6G.
(XLSX)
