# Supplementary figures and images for: Zika virus dysregulates the expression of astrocytic genes involved in neurodevelopment
Source: PLoS Negl Trop Dis. 2021 Apr 23;15(4):e0009362. doi: 10.1371/journal.pntd.0009362 (PMC8099136; doi:10.1371/journal.pntd.0009362)

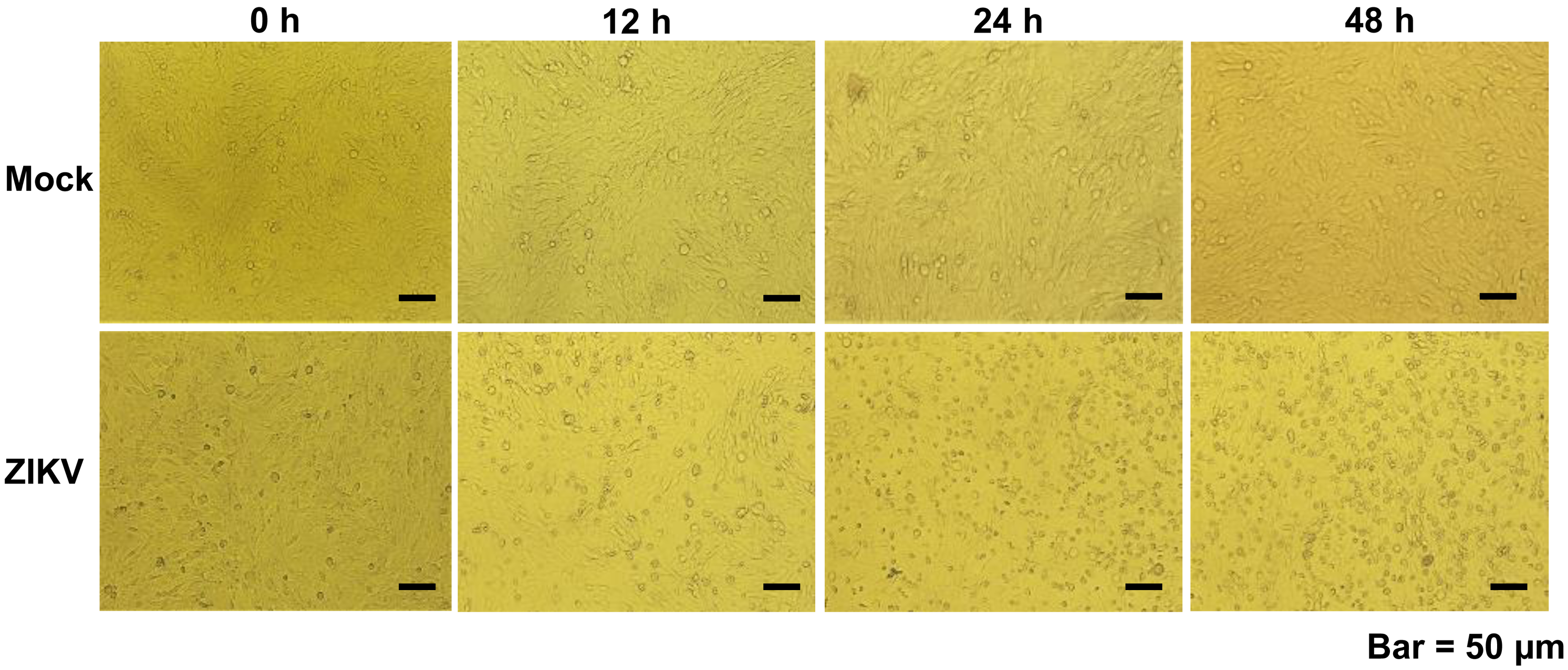

Supplement: S1 Fig — U251 cells were infected with ZIKV at an MOI of 2 for indicated periods. The cytopathic effect (CPE) was displayed in U251 cells infected with ZIKV (bottom) compared to mock (top) with bright-field microscopy, scale bar = 50 μm. (TIF) [file pntd.0009362.s001.tif]
