# Supplementary material for: Zika virus dysregulates the expression of astrocytic genes involved in neurodevelopment
Source: PLoS Negl Trop Dis. 2021 Apr 23;15(4):e0009362. doi: 10.1371/journal.pntd.0009362 (PMC8099136; doi:10.1371/journal.pntd.0009362)
Supplement: S1 Text — (DOCX) [file pntd.0009362.s002.docx]

**S1 Text. Transcriptome Analysis Methodology**

**Transcriptome and bioinformatic analysis**

The RNA-Seq was performed by Illumina Hiseq 2500 PE150 platform (Illumina; San Diego, CA, USA) in Novogene Bioinformatics Institute (Beijing, China).

**RNA quality and quantity**

MPAs were infected with ZIKV at MOI = 2 and harvested at 24 h p.i. In total, four samples were used for RNA-Seq in duplicate, distributed two control samples as Mock group and two ZIKV-infected samples as ZIKV group in MPAs. The total RNA samples were extracted by TRIzol reagent (Invitrogen; Carlsbad, CA, USA), following the manufacturer’s instructions. The quality of extracted RNA was assessed by NanoPhotometer spectrophotometer (IMPLEN, CA, USA). RNA concentration was measured using Qubit®­ RNA Assay Kit in Qubit®2.0 Fluorometer (Life Technologies, CA, USA), and its integrity was assessed using the RNA Nano 6000 Assay Kit of the Bioanalyzer 2100 system (Agilent Technologies, CA, USA).

| **Sample number** | 1 | 2 | 3 | 4 |
| --- | --- | --- | --- | --- |
| **Sample name** | Mock-1# | Mock-2# | ZIKV-1# | ZIKV-2# |
| **Nucleic acid number** | FKRO190063305-1A | FKRO190063306-1A | FKRO190063307-1A | FKRO190063308-1A |
| **Concentration (ng/μl)** | 198 | 206 | 146 | 118 |
| **Volume (μl)** | 42 | 42 | 42 | 42 |
| **Total mass (μg)** | 8.3 | 8.7 | 6.1 | 5.0 |
| **OD260/280** | 1.9 | 1.8 | 1.8 | 1.7 |
| **OD260/230** | 1.8 | 2.0 | 1.6 | 1.4 |
| **RIN** | 8.00 | 7.80 | 6.10 | 7.40 |
| **Test conclusion*** | A | A | A | A |

Note: RIN, RNA Integrity Number; *, the Grade A indicates that the sample quality meets the requirements of database construction and sequencing, and the total quantity could be sufficient in database construction for two or more times.

**Library preparation for Transcriptome sequencing**

A total amount of 3 µg RNA per sample was used as input material for the RNA sample preparations. Sequencing libraries were generated using NEBNext® UltraTM RNA Library Prep Kit for Illumina® (NEB, USA) following manufacturer’s recommendations and index codes were added to attribute sequences to each sample. Briefly, mRNA was purified from total RNA using Poly-T oligo-attached magnetic beads. Fragmentation was carried out using divalent cations under elevated temperature in NEBNext First Strand Synthesis Reaction Buffer (5X). First strand cDNA was synthesized using random hexamer primer and M-MuLV Reverse Transcriptase (RNase H-) (https://www.promega.com/products/pcr/rt-pcr/m-mlv-reverse-transcriptase-rnase-h-minus/). Second strand cDNA synthesis was subsequently performed using DNA Polymerase I and RNase H (https://www.promega.com/). Remaining overhangs were converted into blunt ends via exonuclease/polymerase activities. After adenylation of 3’ ends of DNA fragments, NEBNext Adaptor with hairpin loop structure were ligated to prepare for hybridization. In order to select cDNA fragments of preferentially 250~300 bp in length, the library fragments were purified with AMPure XP system (Beckman Coulter, Beverly, USA). Then 3 µl USER Enzyme (NEB, USA) was used with size-selected, adaptor-ligated cDNA at 37°C for 15 min followed by 5 min at 95 °C before PCR. Then PCR was performed with Phusion High-Fidelity DNA polymerase, Universal PCR primers and Index (X) Primer. At last, PCR products were purified (AMPure XP system) and library quality was assessed on the Agilent Bioanalyzer 2100 system (S2 Text).

**Clustering and sequencing**

The clustering of the index-coded samples was performed on a cBot Cluster Generation System using TruSeq PE Cluster Kit v3-cBot-HS (Illumia) according to the manufacturer’s instructions. After cluster generation, the library preparations were sequenced on an Illumina Hiseq 2500 PE150 platform (Illumina; San Diego, CA, USA) in Novogene Bioinformatics Institute (Beijing, China) (and 125 bp/150 bp paired-end reads were generated).

**Data Analysis**

**Quality control**

Raw data (raw reads) of fastq format were firstly processed through in-house perl scripts. In this step, clean data (clean reads) were obtained by removing reads containing adapter, reads containing Ploy-N and low-quality reads from raw data. At the same time, Q30 and GC content the clean data were calculated. All the downstream analyses were based on the clean data with high quality system (S2 Text).

**Reads mapping to the reference genome**

Reference genome (Genome Reference Consortium Mouse Build 38 [GRCm38 (mm10)])” available in NCBI under the SRA database (BioProject) accession number: PRJNA20689) and gene model annotation files were downloaded from genome website directly. Index of the reference genome was built using Hisat2(v2.0.5) and paired-end clean reads were aligned to the reference genome using Hisat2(v2.0.5) [[1](#_ENREF_1)]. We selected Hisat2 as the mapping tool for that Hisat2 can generate a database of splice junctions based on the gene model annotation file and thus a better mapping result than other non-splice mapping tools system [[1](#_ENREF_1),[2](#_ENREF_2)], and duplicates were removed using Samtools [[3](#_ENREF_3)].

**Quantification of gene expression level**

Feature counts v1.5.0-p3 was used to count the reads numbers mapped to each gene. And then FPKM of each gene was calculated based on the length of the gene and reads count mapped to this gene. FPKM, expected number of Fragments Per Kilobase of transcript sequence per Millions base pairs sequenced, considers the effect of sequencing depth and gene length for the reads count at the same time, and is currently the most commonly used method for estimating gene expression levels. System.

**Differential expression analysis**

Differential expression analysis of two conditions/groups (two biological replicates per condition) was performed using the DESeq2 R package (1.16.1). DESeq2 provide statistical routines for determining differential expression in digital gene expression data using a model based on the negative binomial distribution (https://bioconductor.org/packages/release/bioc/html/DESeq2.html). The resulting P-values were adjusted using the Benjamini and Hochberg’s approach for controlling the false discovery rate. Genes with an adjusted P-value <0.05 found by DEseq2 were assessed as differentially expressed. The gene symbols were converted to ensemble IDs using BioDBnet (https://biodbnet-abcc.ncifcrf.gov/db/dbOrthoRes.php).

The RNA-Seq data was analyzed for the DEGs and then the most significant DEGs were selected on the bases of the highest or lowest fold change and P-values. Then, the top 10 genes were selected on the bases of closely related and important to our designed study, and further analyzed in their RNA and protein levels. Finally, the highly expressed and dysregulated genes were selected and followed for the whole study.

**GO and KEGG enrichment analysis of differentially expressed genes**

Gene Ontology (GO) classification system provides a structured vocabulary to the researchers to know the functional and structural information of a gene. The overall GO structure is arranged in a hierarchical directed acyclic graph (DAG), based on parent (broader) -child (more specialized) terms. The DAG structure is divided into three different GO categories, considering a distinct ontology: Biological Process (BP, the larger processes, or biological programs accomplished by multiple molecular activities), Molecular Function (MF, molecular-level activities performed by gene products), and Cellular Component (CC, the locations relative to cellular structures in which a gene product performs a function) [[4](#_ENREF_4)] .

Gene Ontology (GO) enrichment analysis of differentially expressed genes was implemented by the clusterProfiler R package, in which gene length bias was corrected. GO terms with corrected P-value less than 0.05 were considered significantly enriched by differential expressed genes. The significantly differentially expressed genes selected were subjected to functional annotation analysis (GO) using online ToppGene Suit (https://toppgene.cchmc.org/enrichment.jsp).

KEGG is a database resource for understanding high-level functions and utilities of the biological system, such as the cell, the organism and the ecosystem, from molecular level information, especially large-scale molecular datasets generated by genome sequencing and other high-through put experimental technologies (http://www.genome.jp/kegg/). We used clusterProfiler R package to test the statistical enrichment of differential expression genes in KEGG pathways and DAVID Bioinformatics Resources 6.8 (https://david.ncifcrf.gov/summary.jsp) to find the most related KEGG pathways.

**References**

1. Kim D, Langmead B, Salzberg SL. HISAT: a fast spliced aligner with low memory requirements. Nature methods. 2015;12(4):357-60.

2. Pertea M, Pertea GM, Antonescu CM, Chang T-C, Mendell JT, Salzberg SL. StringTie enables improved reconstruction of a transcriptome from RNA-seq reads. Nature biotechnology. 2015;33(3):290-5.

3. Li H, Handsaker B, Wysoker A, Fennell T, Ruan J, Homer N, et al. The sequence alignment/map format and SAMtools. Bioinformatics. 2009;25(16):2078-9.

4. Dedhia M, Kohetuk K, Crusio WE, Delprato A. Introducing high school students to the Gene Ontology classification system. F1000Research. 2019;8.
