# Supplementary material for: Zika virus dysregulates the expression of astrocytic genes involved in neurodevelopment
Source: PLoS Negl Trop Dis. 2021 Apr 23;15(4):e0009362. doi: 10.1371/journal.pntd.0009362 (PMC8099136; doi:10.1371/journal.pntd.0009362)
Supplement: S2 Text — (DOCX) [file pntd.0009362.s003.docx]

**S2 Text. qRT-PCR Methodology**

**Sample Acquisition, Preparation, and RNA extraction**

MPAs and U251 cells infected with ZIKV for indicated time points were used for qRT-PCR. As sample attainment is the main source of results variability, particularly for RNA targeting experimentations, because mRNA is sensitive as compared to DNA and can be easily perturbed during cells harvesting and processing, so, we deal the samples very carefully in means of harvesting from the cell culture dishes and proceeding for the extraction of RNA. Nucleic acid extraction is another critical step, because variations in tissue or cell-preparation procedures have a significant effect on both RNA quality and yield [[1](#_ENREF_1)]. Total cellular RNA was extracted from cells using TRIzol reagent (Invitrogen; Carlsbad, CA, USA), following the standard protocol provided by the manufacturer. Briefly, 1 mL of TRIzol reagent was added to the cells per 1 well of a 6 well plate, and were isolated using shaker for 2 minutes, and then transferred to 1.5 mL eppendorf tube. To completely dissociate the nucleoproteins complex, cells were vortexed or pipetted for 30 seconds and incubated for 5 minutes at room temperature, and then 0.2 mL of Chloroform (trichloromethane, CHCl3) was added per 1 mL of TRIzol Reagent and vortexed again for 20 seconds. After vertexing, cells were centrifuged at 12,000 × g and 4°C for 10 minutes. The mixture was separated into a colorless upper aqueous phase, interphase, and a lower red phenol-chloroform. The RNA containing colorless aqueous phase was transferred into a new 1.5 mL eppendorf tube, and 70% RNase-free water diluted ethanol was added to an equal volume (1/1) of the aqueous phase, and transferred to column tube containing filter, after shaking up and down 10 times. The tubes were subsequently centrifuged at high speed (12,000 × g) in 4°C for 30 seconds. The filtrate was discarded and the filter was reused to wash the RNAs for 2 times with RNA washing buffers (I and II), according to the manufacturer’s instructions (CWBIO, CW0581M, China). After washing RNAs, empty column tube was centrifuged at high speed for 2 minutes to remove the remaining ethanol drops from the filter which might interfere with RNA and can produce low yield of RNA as a final product. Then, filter was transferred to new RNase-free eppendorf tube and set to dry for 2 minutes to completely evaporate the remaining trace amount of ethanol. 30-50 µl RNase-free water was added to filter containing RNAs and centrifuged at high speed for 1 minute. Finally, the extracted RNA was filtered in eppendorf tube. The RNA concentration was determined using spectrophotometer by the following formula

100 = RNA solution in the tube * conc. of nanodrop / RNA solution in the tube + X

There are some suggestions that firstly, fresh cells or tissues can be put on ice throughout the experiment, to avoid any major effects on RNA concentration and quality, but this belief may be factual for some RNAs and tissues not in every case. Secondly, if RNA samples are not proceeding for experiment immediately, then should be stored at -20, but to avoid any loss it’s better to proceed immediately soon after extraction of RNA [[2](#_ENREF_2)].

**QC of Nucleic Acids**

Extracted samples RNA quantification is critical, because it`s worthwhile to use approximately the same quantity of RNA when comparing different samples. There are numerous common quantification procedures, however, we used spectrophotometry (NanoDrop; Thermo Scientific) (A_260_/A_280_) for the asses the quality and quantity of RNA [[3](#_ENREF_3),[4](#_ENREF_4)]. It is suggested to use a single method only for all samples, to report the information.

**Reverse Transcription**

The reverse-transcription step introduces substantial variation into an RT-qPCR assay [[5](#_ENREF_5),[6](#_ENREF_6)] Complementary DNA (cDNA) was prepared using HiScript II Q Select RT SuperMix, according to the manufacturer’s instructions. Briefly, a total amount of 1 µg RNA per sample was used as input material for the RNA sample preparation. 1 µg RNA was added to 4 µl of HiScript II Q Select RT SuperMix (Vazyme, China) in PCR tube, and raised the final volume to 20 µl with standard grade RNase-free water. The HiScript II Q Select RT SuperMix already contained the random primers, dNTPs, and required enzymes for reverse transcription of RNA into cDNA (<http://www.vazymebiotech.com/products_detail/productId=80.html>). The amplification protocol used as follows: 50°C for 15 minutes, 85°C for 5s, then used for qRT-PCR immediately or be stored at -20℃ for later use.

**qPCR**

Relative quantitative Real-time polymerase chain reaction (qRT-PCR) analysis was performed on Light Cycler 480 (Roche, Basel, Switzerland) using SYBR Green Real-time PCR master mix (Bio-Rad; Hercules, CA, USA) as previously described [[3](#_ENREF_3),[7](#_ENREF_7)]. Briefly, in a reaction mixture of 10 μl SYBR, we added 1 μl cDNA diluted template, 1 μl gene specific primers (10μM, 0.5 μl forward and reverse primer, each), and RNase-free water to complete 20 μl as a final volume. The amplification protocol used as follows: pre-denaturation at 95 °C for 5 min, 40 cycles of 95 °C for 15 s, 60 °C for 15 s, and 72 °C for 30 s, the fluorescence was collected and analyzed at 72 °C step. The melting curve was obtained by heating the amplicon from 50 °C to 95 °C to test primer specificity. The relative expression level was based on 3 biological replicates mean at each time point using the 2^−∆∆CT^ approach [[8](#_ENREF_8)]. *GAPDH* was used as internal reference control in this study.

All the materials e.g., tubes, plates, or plastic strips (manufactures names) were single time used. The plasticwares were clear or white, because the degree of transparency is also important, as different plastics exhibit substantial differences in fluorescence reflection and sensitivity [[9](#_ENREF_9)]. Sealing of the plates were made sure to be tight to avoid evaporation of samples (heat bonding vs adhesives). As PCR efficiency is dependent on the primers used, so we used the primers whose sequences were published or confirmed.

**Data Analysis**

Data analysis includes an evaluation, quality, and reliability of the raw data, and the reportable results generation. Normalization and variability of biological samples was done using 2^−∆∆CT^ approach. It is one of the most reliable way to determine the difference between the concentration of samples, normalized to a single reference gene. GAPDH was used as internal reference gene to normalize the target genes. C_p_ values >30 was suspect and repeated because of the implied low efficiency and generally should not be reported. Amplification efficiency should be determined from the slope of the log-linear portion of the calibration curve. Specifically, PCR efficiency = 10^−1/slope^ − 1, when the logarithm of the initial template concentration (the independent variable) is plotted on the x axis and C_p_ (the dependent variable) is plotted on the y axis. The relative expression level was based on 3 biological replicates mean at each time point using the 2^−∆∆CT^. Briefly, the raw data from the machine was transferred to excel file and then the Cp value of target gene (in triplicate) was subtracted from the average (mean) of the Cp values of reference gene (GAPD). Secondly, the ∆Cp value of calculated result from previous step was determined using power 0.5. Then the average of the ∆Cp values (triplicate) was calculated and followed the fold change determination by dividing ∆Cp value of each well (triplicate) by the average of ∆Cp values. Statistical significance was determined by paired Student’s *t*-test using the Prism 8 software (GraphPad Software Inc., San Diego, CA, USA). A *P* ≤ 0.05 was considered statistically significant.

**References:**

1. Morrogh M, Olvera N, Bogomolniy F, Borgen PI, King TA. Tissue preparation for laser capture microdissection and RNA extraction from fresh frozen breast tissue. Biotechniques. 2007;43(1):41-8.

2. Micke P, Ohshima M, Tahmasebpoor S, Ren Z-P, Östman A, Pontén F, et al. Biobanking of fresh frozen tissue: RNA is stable in nonfixed surgical specimens. Laboratory investigation. 2006;86(2):202-11.

3. Bustin SA, Benes V, Garson JA, Hellemans J, Huggett J, Kubista M, et al. The MIQE Guidelines: M inimum I nformation for Publication of Q uantitative Real-Time PCR E xperiments. Oxford University Press; 2009.

4. Nolan T, Hands RE, Bustin SA. Quantification of mRNA using real-time RT-PCR. Nature protocols. 2006;1(3):1559-82.

5. Stahlberg A, Hakansson J, Xian X, Semb H, Kubista M. Properties of the reverse transcription reaction in mRNA quantification. Clinical chemistry. 2004;50(3):509-15.

6. Stahlberg A, Kubista M, Pfaffl M. Comparison of reverse transcriptases in gene expression analysis. Clinical chemistry. 2004;50(9):1678-80.

7. Luo Z, Su R, Wang W, Liang Y, Zeng X, Shereen MA, et al. EV71 infection induces neurodegeneration via activating TLR7 signaling and IL-6 production. PLoS pathogens. 2019;15(11):e1008142.

8. Livak KJ, Schmittgen TD. Analysis of relative gene expression data using real-time quantitative PCR and the 2− ΔΔCT method. methods. 2001;25(4):402-8.

9. Reiter M, Pfaffl MW. Effects of plate position, plate type and sealing systems on real-time PCR results. Biotechnology & Biotechnological Equipment. 2008;22(3):824-8.
